# Decoding the infrastructure of the cerebellum

**DOI:** 10.7554/eLife.60852

**Published:** 2020-08-19

**Authors:** Willem S van Hoogstraten, Chris I De Zeeuw

**Affiliations:** 1 Department of Neuroscience, Erasmus MCRotterdamNetherlands; 2 Netherlands Institute for Neuroscience, NIN-KNAWAmsterdamNetherlands

**Keywords:** zebrin, aldolase C, arousal, autonomic, brain, cerebellar cognitive affective syndrome, Mouse

## Abstract

High-end technical approaches help to untangle the substructure and projection patterns of the cerebellum.

**Related research article** Fujita H, Kodama T, du Lac S. 2020. Modular output circuits of the fastigial nucleus for diverse motor and nonmotor functions of the cerebellar vermis. *eLife* **9**:e58613. doi: 10.7554/eLife.58613

Our brains must constantly juggle and combine a multitude of daily tasks, such as talking while walking, or planning our next move. These seemingly mundane actions rely on complex brain networks that interact through anatomical hubs formed of several types of cells. For example, a brain structure called the cerebellum is connected to various networks in the lower and higher brainstem, which it uses to help coordinate conscious and unconscious movements as well as cognitive processes like decision-making (Gao et al., 2018; Chabrol et al., 2019). The cerebellum is divided into a series of compartments known as cerebellar nuclei, which are split into multiple groups of cells (Teune et al., 2000). Some of these cell groups have overlapping or related roles, making it difficult to determine which structures in the cerebellum are linked to specific tasks (Romano et al., 2020).

For instance, the medial and lateral cerebellar output nuclei, which share many anatomical targets, show both similarity and differences in their connections (or ‘projections’) to these sites (Middleton and Strick, 1997; Teune et al., 2000). Indeed, recent physiological studies suggest that these medial and lateral compartments, respectively, play a role in simple and complex forms of motor planning (Gao et al., 2018; Chabrol et al., 2019). However, both studies used manipulations that were not cell-specific, making it difficult to establish detailed conclusions on the origin of control. This illustrates why it is important to disentangle how individual groups of cells within the two nuclei connect to downstream brain networks involved in planning actions. Now, in eLife, Hirofumi Fujita, Sascha du Lac and Takashi Kodama from Johns Hopkins University report a new cell-specific approach, presenting the most comprehensive, functional connectivity study of any cerebellar nucleus to date (Fujita et al., 2020).

The team used single-cell gene expression analysis and immunohistochemistry to explore the different groups of cells present in the medial cerebellar nucleus of mice. This revealed five distinct subgroups of cells: four groups differed based on molecular expression patterns, including one which could be split into two further subgroups based on anatomical location.

Next, Fujita et al. carried out a series of tracer experiments to map how each of the five identified subgroups was connected to different areas of the brain. This approach used viral transneuronal tracers, which exploit the ability for certain viruses to ‘jump’ across the junction that connects two neurons. The resulting input-output maps were nearly completely segregated. This highlighted that each subgroup in the nucleus had divergent projection patterns and was anatomically connected to separate, large-scale networks that play different roles in voluntary or involuntary ‘autonomic’ functions (Figure 1). The constitution of these networks suggest that some may be predisposed to transmit fast signals, while others transmit signals more slowly. This is a crucial step for understanding how different cell groups in the medial nucleus may play specific roles, and how they may work together to integrate different types of responses (Romano et al., 2020).

**Figure 1.**
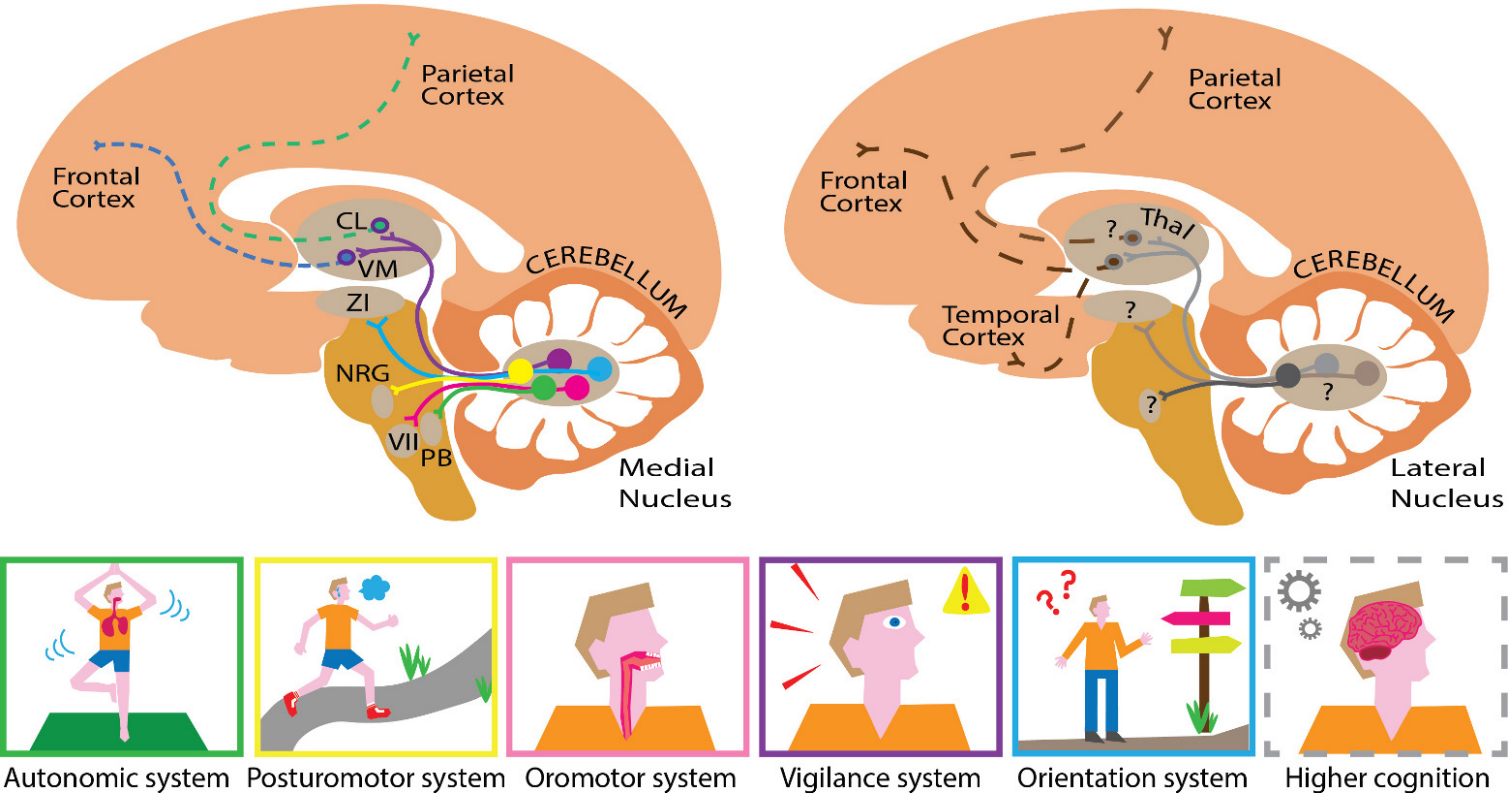
Mapping the substructures and projections of the medial and lateral cerebellar nuclei. Five different cell groups can be identified in the medial cerebellar nucleus (left). Each projects to a specific downstream network of targets, which serve a set of related functions (bottom panel). For example, cells that project to the zona incerta (ZI; blue pathway) help control orientation, while cells that project to the nucleus reticularis gigantocellularis (NRG; yellow pathway) are involved in regulating posture. The projections highlighted here form only a small part of the actual elaborate networks shown by Fujita and colleagues. This approach could also be used to elucidate pathway-specific cell groups in the lateral cerebellar nucleus (right). This compartment presumably projects to similar parts of the cerebral cortex (dashed lines) through different hubs that probably serve higher cognitive functions. This illustrates how the medial and lateral cerebellum might complement each other, targeting similar, but distinct hubs that relay signals to partially overlapping areas in the brain. The cortex is shown in light orange, the cerebellum in dark orange, the brainstem in mustard and the thalamus (Thal) in light brown. CL refers to the centrolateral nucleus of the thalamus, PB to the parabrachial nucleus, VM to the ventromedial nucleus of the thalamus, and VII to the facial motor nucleus.

Image credit: Chris I. De Zeeuw, Willem S. van Hoogstraten and Valentina Riguccini (CC BY 4.0).

There are, however, potential caveats associated with the individual high-end technical methods harnessed in this study. For example, the approaches used to genetically engineer the labels used in the viral transneuronal tracing experiments may allow some neurons to be tagged by accident, and for brain cells to be misidentified as being part of the output network the nucleus connects to (Sjulson et al., 2016; Song and Palmiter, 2018; Zingg et al., 2017). It was therefore reassuring that Fujita et al. used multiple approaches to confirm their major high-tech observations, and that they only reported projections previously identified by conventional tracing. These decisions reduced the likelihood of false-positive interpretations – that is, incorrectly reporting neurons as belonging to the network.

Similarly, further experiments could also be conducted to avoid potential false-negative labelling – failing to report neurons which connect to subgroups in the medial cerebellar nucleus. In particular, it could be worthwhile to dedicate another line of transneuronal tracing experiments to the hubs in the rest of the brain that the medial cerebellar cell groups connect to. These downstream nuclei display widespread connectivity to other parts of the brain, suggesting that specific cell groups in the medial cerebellar nucleus connect to other networks through particular second-order neurons in these hubs (Wang et al., 2020).

Identifying genetically distinct groups of neurons, combined with elucidating their specific projection networks, may well pave the way for new breakthroughs. For instance, this could be used as a roadmap to alter the function of specific cell groups in the medial nucleus as animals perform tasks of interest. Moreover, the same genetically-driven approach deployed by Fujita et al. could help to identify different subgroups within the lateral cerebellar nucleus, allowing direct functional comparisons with the medial nucleus (Figure 1). This would help to understand the extent to which specific cell groups in the medial and lateral cerebellum overlap or complement one another in controlling autonomic, sensorimotor or cognitive functions (Gao et al., 2018; Chabrol et al., 2019; Romano et al., 2020).

In addition to showing how to alter specific cell types in the medial nucleus at a high spatial resolution, Fujita et al. reveal how to manipulate these cells over time. Their work highlights the proteins required for signals to be transduced quickly or slowly, and it connects the nuclei neurons that express these proteins to fast or slow inhibitory cell inputs. Ultimately, this provides all the knowledge needed to design meaningful functional experiments, offering a bewildering palette of insight that should inspire neuroscientists for many years to come.
